# Public Transportation and Tuberculosis Transmission in a High Incidence Setting

**DOI:** 10.1371/journal.pone.0115230

**Published:** 2015-02-23

**Authors:** Carlos Zamudio, Fiorella Krapp, Howard W. Choi, Lena Shah, Antonio Ciampi, Eduardo Gotuzzo, Jody Heymann, Carlos Seas, Timothy F. Brewer

**Affiliations:** 1 Instituto de Medicina Tropical Alexander von Humboldt, Universidad Peruana Cayetano Heredia, Lima, Peru; 2 Department of International Health, Bloomberg School of Public Health, Johns Hopkins University, Baltimore, United States of America; 3 Department of Epidemiology, Biostatistics & Occupational Health, McGill University. Montreal, Canada; 4 Departamento de Enfermedades Infecciosas, Tropicales y Dermatologicas, Hospital Nacional Cayetano Heredia, Lima, Peru; 5 Department of Epidemiology, Jonathan and Karin Fielding School of Public Health, University of California Los Angeles, Los Angeles, United States of America; 6 Department of Medicine, David Geffen School of Medicine, University of California Los Angeles, Los Angeles, United States of America; St. Petersburg Pasteur Institute, RUSSIAN FEDERATION

## Abstract

**Background:**

Tuberculosis (TB) transmission may occur with exposure to an infectious contact often in the setting of household environments, but extra-domiciliary transmission also may happen. We evaluated if using buses and/or minibuses as public transportation was associated with acquiring TB in a high incidence urban district in Lima, Peru.

**Methods:**

Newly diagnosed TB cases with no history of previous treatment and community controls were recruited from August to December 2008 for a case-control study. Crude and adjusted odd ratios (OR) and 95% confidence intervals (CI) were calculated using logistic regression to study the association between bus/minibus use and TB risk.

**Results:**

One hundred forty TB cases and 80 controls were included. The overall use of buses/minibuses was 44.9%; 53.3% (72/135) among cases and 30.4% (24/79) among controls [OR: 3.50, (95% CI: 1.60–7.64)]. In the TB group, 25.7% (36/140) of subjects reported having had a recent household TB contact, and 13% (18/139) reported having had a workplace TB contact; corresponding figures for controls were 3.8% (3/80) and 4.1% (3/73), respectively[OR: 8.88 (95% CI: 2.64–29.92), and OR: 3.89 (95% CI: 1.10–13.70)]. In multivariate analyses, age, household income, household contact and using buses/minibuses to commute to work were independently associated with TB [OR for bus/minibus use: 11.8 (95% CI: 1.45–96.07)].

**Conclusions:**

Bus/minibus use to commute to work is associated with TB risk in this high-incidence, urban population in Lima, Peru. Measures should be implemented to prevent TB transmission through this exposure.

## Background

Peru has one of the highest incidence rates of tuberculosis (TB) in the Americas [1] despite having a well-functioning National TB Program (NTP) that regularly meets recommended World Health Organization (WHO) [2] targets. As per WHO guidelines, TB control strategies in Peru have focused on successful treatment of active TB patients and targeted case-finding in high-risk groups such as pediatric household contacts of smear-positive patients [3,4] However, molecular epidemiologic methods demonstrate that in high-burden populations extra-domiciliary exposure clearly plays a role in TB transmission even among patients with known household contacts [5]. Though extra-domiciliary TB transmission happens, few studies have evaluated the magnitude of this transmission or the venues in which this transmission occurs in TB endemic settings.

TB transmission while using public transportation has been reported [6–9]. Recent studies also suggest that using public transportation (buses/minibuses) in Lima is associated with an increased risk of TB [10–12]; if correct, these findings may have significant public health implications given the reliance of large segments of the population on buses/minibuses to commute in Lima and other major cities in TB endemic areas. These studies were limited however by a lack of appropriate comparison groups, a limited ability to control for other potential TB risk factors, or both [10–12]. We therefore undertook this study to evaluate the possible role of bus/minibus use on TB risk in Lima.

## Methods

### Study Setting

San Juan de Lurigancho (SJL) is the most densely populated district of Lima with a population of 898,443 inhabitants spread over 131.25 km^2^, resulting in a population density of 6,850 inhabitants/km^2^ [13]. The SJL district is one the poorest districts in Peru; 62.5% of the residents have an income of less than $50 USD per month.[13] This district has 33 peripheral health care centers and one referral hospital, each of which has a TB unit to provide TB treatment following NTP guidelines. In 2007, SJL accounted for 3.2% of the Peruvian population but reported 7.0% (2,044/29,393) of all TB cases and 14.2% (116/818) of all MDR-TB cases [4].

### Study description

A case-control study to identify transportation use risk factors for primary TB in SJL was conducted. The protocol has been previously published in detail, but briefly cases and controls were recruited between August 1, 2008 and December 12, 2008 in the SJL district of Lima [14]. All adult patients ≥ 18 years old with laboratory proven MDR-TB, no history of previous TB treatment and who were being followed in a SJL NTP clinic during the recruitment period were eligible to be cases. Eighty drug-sensitive pulmonary TB cases were randomly selected from a database of all drug-sensitive TB patients followed in SJL NTP clinics; medical records were reviewed to determine eligibility, which included ≥ 18 years old, no history of previous TB treatment, sputum smear-negative at 2 months on NTP standard therapy and no treatment failure or relapse at the time of interview. To select controls, a population weighted sampling method based on the 34 NTP clinic catchment areas covering SJL was developed. A computer-generated randomization scheme was used to select places within catchment areas where research nurses went to find controls. Upon arriving at the randomly selected locations, the first adult ≥ 18 year old encountered and who on questioning did not have symptoms suggestive of active TB was invited to participate in the study. If that person refused or was not eligible, the interviewer approached another person until someone agreed to participate [14]. All potential controls were screened for cough for > 14 days and other TB symptoms; subjects who reported any TB symptoms were not included as controls and were referred to a health center. All study participants provided informed, written consent before enrollment.

### Data Source

Questionnaires were developed to explore demographic, socioeconomic and behavioral factors associated with TB. Information about income, education, transportation, housing conditions, employment, neighborhood characteristics, prison exposure, health and personal behaviors were elicited. Development and validation of the questionnaire is detailed in the original article [14]. The transportation section of the questionnaire recorded the type and commute time of transportation used to go to work and to health care centers. Traveling to work was the most frequent reason for using transportation among cases and controls. Transportation use was categorized by bus/minibus use, other (mototaxi, taxi, private car or walking), or working at home/unemployed. For cases, questions regarding transportation and healthcare use, employment and income referred to the three years prior to TB diagnosis; for controls, the reference period was the three years prior to interview.

### Statistical methods

After descriptive analysis, potential TB risk factors were assessed using logistic regression. Study variables (types of transportation used to go to work or the clinic/hospital) and all other variables associated with TB risk in the univariate analysis with a p value ≤ 0.1 were included in a backward logistic regression analysis. Crude and adjusted ORs with 95% confidence intervals (CI) were calculated. Generalized lineal model was used to better estimate ORs in this settings of high prevalence of bus/minibus usage. The ORs did not differ with this analysis and were not further considered.

### Ethical review

The study was reviewed and approved by the Human Subjects Review Committees of Universidad Peruana Cayetano Heredia (UPCH), the McGill University Health Centre Research Institute and the Ministry of Health Dirección de Salud Lima Este, Peru.

## Results

One hundred forty TB cases and 80 community controls were included in the study; 53.2% of participants were males (117/220) and 47.3% were 25–50 years old (Table 1). There was no difference between cases and control by gender, but TB cases were significantly younger [OR 0.36 (95% CI: 0.16–0.84)], less likely to be married [OR 0.27 (95% CI: 0.12–0.60)] and had less household income [OR 0.35 (95% CI: 0.15–0.81)] than controls (Table 1).

**Table 1:** Bivariate assessment of risk factors for tuberculosis.

Variables	Community control (n = 80) N (%)	TB Cases (n = 140) N (%)	Crude OR	95% Conf. Interval	p
Gender					
Male	42 (52.5)	75 (53.6)	1.00	ref	NA
Female	38 (47.5)	65 (46.4)	0.96	0.55–1.66	0.878
Marital status					
Single	25 (31.3)	67 (47.9)	1.00	ref	NA
Living together	29 (36.3)	43 (30.7)	0.55	0.29–1.07	0.078
Married	21 (26.3)	15 (10.7)	0.27	0.12–0.60	0.001
Divorced	2 (2.5)	12 (8.6)	2.24	0.47–10.72	0.313
Widow	3 (3.8)	3 (2.1)	0.37	0.07–1.97	0.246
Age					
< 25 years old	19 (23.8)	62 (44.3)	1.00	ref	NA
25–50 years old	45 (56.3)	59 (42.1)	0.40	0.21–0.76	0.006
> 50 years old	16 (20)	19 (13.6)	0.36	0.16–0.84	0.018
Electricity in house					
No	1 (1.3)	7 (5.0)	1.00	ref	NA
Yes	79 (98.8)	132 (95.0)	0.24	0.03–1.98	0.184
Monthly household income					
< 550 soles	23 (31.1)	58 (48.3)	1.00	ref	NA
550–1000 soles	33 (44.6)	46 (38.3)	0.55	0.29–1.07	0.077
> 1000 soles	18 (24.3)	16 (13.3)	0.35	0.15–0.81	0.014
Number of people living in household					
Zero	2 (2.5)	6 (4.3)	1.00	ref	NA
1–2 persons	13 (16.3)	26 (18.6)	0.67	0.12–3.77	0.647
3–4 persons	24 (30.0)	36 (25.7)	0.50	0.09–2.69	0.419
5–6 persons	22 (27.5)	30 (21.4)	0.45	0.08–2.47	0.361
7–8 persons	14 (17.5)	16 (11.4)	0.38	0.07–2.20	0.281
> 8 persons	5 (6.3)	26 (18.6)	1.73	0.27–11.19	0.563
Transportation to health center					
Did not use health center	17 (21.3)	34 (24.3)	1.00	ref	NA
Bus/minibus	41 (51.3)	51 (36.4)	0.62	0.30–1.27	0.192
Other	22 (27.5)	55 (39.3)	1.25	0.58–2.68	0.567
Duration living in same house (months, by quartiles)					
1st quartile (shortest duration)	19 (23.8)	38 (27.5)	1.00	ref	NA
2nd quartile	18 (22.5)	34 (24.6)	0.94	0.43–2.09	0.888
3rd quartile	23 (28.8)	37 (26.8)	0.80	0.38–1.72	0.573
4th quartile (longest duration)	20 (25.0)	29 (21.0)	0.73	0.33–1.60	0.426
Duration working at same place (years, by quartiles)					
1st quartile (shortest duration)	29 (36.7)	26 (19.3)	1.00	ref	NA
2nd quartile	17 (21.5)	54 (40.0)	3.54	1.66–7.57	0.001
3rd quartile	15 (19.0)	27 (20.0)	2.01	0.88–4.58	0.097
4th quartile (longest duration)	18 (22.8)	28 (20.7)	1.74	0.78–3.84	0.174
Transportation to workplace					
Work at home /unemployed	21 (26.6)	18 (13.3)	1.00	ref	NA
Bus/minibus	24 (30.4)	72 (53.3)	3.50	1.60–7.64	0.002
Other	34 (43.0)	45 (33.3)	1.54	0.71–3.34	0.270
Commute time to work					
Work at home /unemployed	22 (34.4)	19 (14.4)	1.00	ref	NA
≤ 60 minutes	34 (53.1)	78 (59.1)	2.66	1.27–5.54	0.009
> 60 minutes	8 (12.5)	35 (26.5)	5.07	1.90–13.54	0.001
Commute time to health center					
Did not go to health center	17 (21.3)	34 (24.3)	1.00	ref	NA
≤ 20 minutes	38 (47.5)	66 (47.1)	0.87	0.43–1.76	0.695
> 21 minutes	25 (31.3)	40 (28.6)	0.80	0.37–1.72	0.569
Hospital visits frequency					
<1 per year	29 (36.3)	42 (30.0)	1.00	ref	NA
1–4 per year	40 (50.0)	72 (51.4)	1.24	0.67–2.29	0.486
>5 per year	11 (13.8)	26 (18.6)	1.63	0.70–3.81	0.258
Household contact with tuberculosis					
No	77 (96.3)	104 (74.3)	1.00	ref	NA
Yes	3 (3.8)	36 (25.7)	8.88	2.64–29.92	<0.001
Workplace contact with tuberculosis					
No / do not know	68 (93.2)	105 (75.5)	1.00	ref	NA
Yes	3 (4.1)	18 (13.0)	3.89	1.10–13.70	0.035
Work at home /unemployed	2 (2.7)	16 (11.5)	5.18	1.15–23.25	0.032

Fifty-three percent of TB cases (72/135) used a bus/minibus to commute to work compared with 30.4% (24/79) of controls [OR 3.5 (95% CI: 1.60–7.64)]. The commute time to work, regardless of the method used, also was associated with TB risk in a dose response fashion. The risk of TB increased 2.66 fold (95% CI: 1.27–5.54) for individuals with commute times ≤ 60 minutes, and over 5 fold for persons with commute times > 60 minutes [OR 5.07 (95% CI: 1.90–13.54)]. TB cases were more likely to report having had a recent household contact with TB than community controls, 25.7% (36/140) versus 3.8% (3/80), OR 8.88 (95% CI: 2.64–29.92), as well as having had a workplace contact with TB, 13% (18/139) versus 4.1% (3/73), OR 3.89, (95% CI: 1.10–13.70) (Table 1).

In the multivariate analysis, older age and higher household income remained protective against acquiring TB, with adjusted ORs of 0.19 (95% CI: 0.04–0.97) for age > 50 years old, and 0.18 (95% CI: 0.05–0.62) for a monthly household income higher than one thousand soles (approximately $300 USD), respectively. Recent household contact with an active TB patient remained independently associated with TB risk, adjusted OR of 26.37 (95% CI: 3.98–174.72), as was being divorced, adjusted OR 7.11 (95% CI: 1.00–50.44). Using a bus/minibus to commute to work also was independently associated with the risk of having TB, with an adjusted OR of 11.8 (95% CI: 1.45–96.07) (Table 2).

**Table 2:** Multivariate assessment of risk factors for tuberculosis.

Variables	Adjusted OR	95% Conf. Interval	p
Marital status			
Single	1.00	ref	NA
Living together	1.00	0.35–2.86	0.996
Married	0.73	0.18–3.02	0.666
Divorced	7.11	1.00–50.44	0.05
Widow	0.88	0.05–14.31	0.929
Age			
< 25 years old	1.00	ref	NA
25–50 years old	0.41	0.14–1.20	0.104
> 50 years old	0.19	0.04–0.97	0.046
Monthly household income			
< 550 soles	1.00	ref	NA
550–1000 soles	0.51	0.19–1.36	0.176
> 1000 soles	0.18	0.05–0.62	0.006
Transportation to health center			
Did not go to health center	1.00	ref	NA
Bus/minibus	0.76	0.24–2.43	0.646
Other	3.00	0.92–9.69	0.068
Duration working at same place (years, by quartiles)			
1st quartile (shortest duration)	1.00	ref	NA
2nd quartile	3.14	0.60–16.54	0.177
3rd quartile	3.09	0.52–18.39	0.215
4th quartile (longest duration)	3.16	0.55–18.11	0.197
Transportation to workplace			
Work at home /unemployed	1.00	ref	NA
Bus/minibus	11.8	1.45–96.07	0.021
Other	3.47	0.40–30.28	0.261
Household contact with tuberculosis			
No	1.00	ref	NA
Yes	26.37	3.98–174.72	0.001
Workplace contact with tuberculosis			
No / do not know	1.00	ref	NA
Yes	2.43	0.43–13.56	0.312
Work at home /unemployed	drop		

## Discussion

In this urban Lima population with high rates of TB, using buses/minibuses to commute to work was independently associated with having active TB even after controlling for other known risk factors such as household contact, socioeconomic factors and age. Regular buses/minibuses users were almost 12 times more likely to have TB than non-users. Time commuting to work was not associated with TB in the multivariate analysis, suggesting that the type of transportation used may be contributing to the dose response trend observed in the univariate analysis. These results also build on previous studies suggesting a relationship between using buses/minibuses in Lima and risk for TB by controlling for other known TB risk factors [10–12].

TB transmission through casual contact in extra-domiciliary settings has been documented [15], including while using public transportation such as buses and airplanes [6–9,15–18]. While these studies demonstrate that TB transmission can occur when using public transportation systems and that the risk of transmission increases with proximity to the index case and the duration of exposure, they have not provided an estimate of the contribution of a public transportation system use to overall risk in a TB endemic population.[16] Given the millions of individuals living in TB endemic regions around the world who use buses/minibuses for public transportation daily, even slight increases in TB risk could have substantial public health implications.

Numerous factors may contribute to an increased TB risk from regularly using bus/minibus systems in this high-risk setting. Buses and minivans in SJL are overcrowded and poorly ventilated, providing ample opportunity for transmission of airborne pathogens. This District has a high population density, significant air pollution, a congested road network and an insufficient and poorly organized public transportation system [19]. These factors, combined with among the highest TB rates in Peru and Latin America, mean that there are ample opportunities for TB transmission to occur while using buses/minibuses.

Our study has several strengths. These strengths include interviewing randomly selected controls who live and work in the same district as cases, being able to control for other known TB risk factors including socioeconomic status, known TB exposures at home and in the workplace, human immunodeficiency virus status, crowding, and age as well as having quantitative data on bus/minibus use. Recall bias and misclassification bias are potential limitations of case-controls studies. Commuting to work in our population is a common daily activity; there is no reason to suspect that TB cases would be more likely to recall using buses/minibuses than controls. TB cases were drawn from patients confirmed by acid-fast bacilli smear or culture; controls were screened for TB symptoms. Cases and controls could have used buses/minibuses for trips besides traveling to work or health facilities, but we assume that bus/minibus use to commute to work is a good proxy for overall bus/minibus use. Hence, misclassification, if it did occur, should serve to reduce the likelihood of a difference between the two groups. Exposure to TB contacts in the workplace was associated with TB risk in the univariate, but not the multivariate analyses. When workplace exposure and buses/minibuses use were added to the multivariate model, workplace exposure dropped out as a significant risk factor for TB. As another limitation, it is possible that some of the increased risk of TB being attributed to bus/minibus use to commute to work may be due to TB exposure in the workplace, but we cannot distinguish further between these two possible effects with the available data. Additional studies may help clarify an interaction, if any, between these two risk factors. Being divorced also was associated with a higher risk of TB; it is possible that being chronically ill or having TB leads to higher divorce rates rather than the reverse.

Much of the world’s growth in urbanization, and hence need for increasing buses/minibuses use, is occurring in TB endemic regions such as the greater Lima metropolitan area. Though TB transmission using public transportation happens [10–12,20,21], there are no clear recommendations regarding what steps should be taken in TB endemic areas, if any, to reduce this risk [16,22]. Our results fill a crucial gap in this debate by demonstrating, at least in SLJ, that using buses/minibuses is a substantial risk factor for acquiring TB even after controlling for other known risk factors. These data suggest that as urban populations grow and bus/minibus use expands in areas with TB risks similar to SLJ, public health officials should be aware that public transportation use may be an important contributor to TB transmission in these settings.
